# Microbiomes in the insectivorous bat species *Mops condylurus* rapidly converge in captivity

**DOI:** 10.1371/journal.pone.0223629

**Published:** 2020-03-20

**Authors:** Kathryn M. Edenborough, Andre Mu, Kristin Mühldorfer, Johanna Lechner, Angelika Lander, Marcel Bokelmann, Emmanuel Couacy-Hymann, Aleksandar Radonic, Andreas Kurth

**Affiliations:** 1 Centre for Biological Threats and Special Pathogens, Robert Koch Institute, Berlin, Germany; 2 Department of Microbiology & Immunology, Microbiological Diagnostic Unit Public Health Laboratory at the Peter Doherty Institute, University of Melbourne, Melbourne, Australia; 3 Department of Wildlife Diseases, Leibniz Institute for Zoo and Wildlife Research, Berlin, Germany; 4 Methodology & Research Infrastructure, Robert Koch Institute, Berlin, Germany; 5 LANADA, Laboratoire National d’Appui au Développement Agricole, Bingerville, Cote d’Ivoire; University of Minnesota Twin Cities, UNITED STATES

## Abstract

Bats are well known reservoir hosts for RNA and DNA viruses. The use of captive bats in research has intensified over the past decade as researchers aim to examine the virus-reservoir host interface. In this study, we investigated the effects of captivity on the fecal bacterial microbiome of an insectivorous microbat, *Mops condylurus*, a species that roosts in close proximity to humans and has likely transmitted viral infections to humans. Using amplicon 16S rRNA gene sequencing, we characterized changes in fecal bacterial community composition for individual bats directly at the time of capture and again after six weeks in captivity. We found that microbial community richness by measure of the number of observed operational taxonomic units (OTUs) in bat feces increases in captivity. Importantly, we found the similarity of microbial community structures of fecal microbiomes between different bats to converge during captivity. We propose a six week-acclimatization period prior to carrying out infection studies or other research influenced by the microbiome composition, which may be advantageous to reduce variation in microbiome composition and minimize biological variation inherent to *in vivo* experimental studies.

## Introduction

Bats (*Mammalia*, *Chiroptera)* play an important role in pollination and pest control [1] and are natural host reservoirs for many RNA and DNA viruses known to cause significant morbidity and mortality in humans [2]. To effectively study their role as reservoir hosts for pathogenic viruses, bats have been housed in captivity [3–6]. Introduction of bats to captive environments entails dietary adaptation, increased stress and habitat change [7,8]. The captive environment indirectly modifies the microbiome with respect to the number of microbial species in the community (alpha diversity) and the phylogenetic similarity of the microbial communities between separate bats (beta diversity) [9,10].

Enteric bacterial community structure is known to modulate viral infection severity in insects and mammals (reviewed in [11] and [12]) and has been shown to either promote or restrict infection for poliovirus [13], norovirus [14] and influenza [15]. In laboratory studies, infection is adversely influenced by microbiome variation between cages (cage effects), a factor identified as a source of extraneous variation that can lead to incongruent results (reviewed in [16]). Therefore, generating an acclimatized microbiome in animals captured from the wild may be important to reduce experimental variation in disease outcomes, severity of virus infection and virulence.

The fecal microbiomes in individual wild bats are unique and cluster based on host phylogeny and feeding strategy [17], however in captivity the microbial communities have been shown to converge over six months [9]. Convergence to shared microbial communities by the captive group is largely influenced by diet (based on phylogenetically aware diversity metrics such as UniFrac). For captive studies, bats are typically introduced into captivity over time and therefore the individuals originate from different geographical locations and are likely exposed to regional diets. Identifying a minimum period of housing for the microbiome to converge in captivity would assist researchers to choose more accurate acclimatization periods to prevent the influence of inter-animal variation of the microbiome from confounding experimental findings.

Our current knowledge of microbial convergence of the fecal microbiome in captive bats extends to a six-month window and we therefore sought to measure changes to the fecal microbiome in captivity within a shorter time frame. We also focused on the bat species *Mops condylurus (M*. *condylurus)* that roosts in close proximity to villages and has likely transmitted viral infections to humans in the past [18]. Fecal samples were collected from insectivorous bats prior to capture (pre-capture samples) and following six weeks in captivity (post-capture samples) and using amplicon 16S rRNA gene sequencing to interrogate the microbiota, we found the microbial communities in bats became phylogenetically similar within six weeks of captivity.

## Materials and methods

### Sample collection

Animal capture, handling and sampling were performed with the permission of the Laboratoire Central Vétérinair, Laboratoire National D’Appui Au Developpement Agricole (LANADA), Bingerville, Ivory Coast according to the approved animal ethics application (No. 05/Virology/2016). In this application, the animal care and use protocol was approved by the Institutional Animal Care and Use Committee of LANADA and the National Ethics Committee of the Research (CNER). Consent existed to capture the bats from the owners of the residence in Koffikro Village. *Mops condylurus* bats were captured with mist nets near Koffikro, Ivory Coast, and held individually in cotton bags until transfer to captivity at the Le Laboratoire Central Vétérinaire de Bingerville (LCVB). Bats were held in a steel framed meshed aviary, sized 5 metres in length and 4 metres in width. Roosting boxes and hanging soft material were provided as hiding places for the bats. The captive diet consisted of mealworms (*Tenebrio molitor*) which were fed to bats with tweezers or were first made into a mealworm puree and fed to bats via syringe until the point of satisfaction. Fecal samples were collected twice; directly after capture (pre-capture) and at six weeks of captivity (post-capture) for microbial community profiling using amplicon 16S rRNA gene sequencing. Samples were obtained by collection of fecal pellets (~100–300 mg) in screw cap 2 ml micro tubes (Sarstedt) from cotton bags used to hold individual microbats for about two hours. After collection fecal samples were frozen at -80°C and later transported to the Robert Koch Institute, Berlin in a cryogenic dry shipper where they were stored at -80°C until genomic DNA extraction.

### Metadata for microbiome datasets

Fecal pellets were collected directly from 20 individual *M*. *condylurus* bats upon capture (herein referred to as pre-capture samples) and from the same animal six weeks after introduction to captivity (herein referred to as post-capture samples) following adaptation to the feeding program. There was sufficient fecal material to perform nucleic acid extractions for nine pre-capture and 18 post-capture bats. All bats were species confirmed as *M*. *condylurus* by amplification and sequencing of the cytochrome b gene. The metadata for these samples is shown in S1 Table.

### Nucleic acid extraction

Genomic DNA was extracted from ~100 mg of feces with the use of a Nucleospin DNA Stool Kit (740472, Macherey-Nagel) according to the manufacturer’s instructions with the following modifications; for sample preparation each Nucleospin Tube Type A containing 100 mg of feces was loaded into pre-chilled adapters and shaken twice for 30 sec with a TissueLyser II instrument (Qiagen) at a frequency of 25 Hz. Following lysis at 70°C for 5 mins, the samples were further disrupted by vigorous shaking with a ThermoMixer (Eppendorf) at room-temperature, 2000 rpm for 10 min. Nucleic acids were eluted in 50 μl elution buffer and stored at -20°C.

### Small Subunit 16S rRNA gene V3-V4 amplification with fusion primers

Amplification and purification of V3-V4 regions of the 16S rRNA genes were performed according to the 16S Metagenomic Sequencing Library Preparation protocol [19] from Illumina ^®^ with the following modifications; PCR was carried out with Kapa High Fidelity (HiFi) PCR Kit (KK2101, Kapa Biosystems) by the addition of 25 ng DNA to a master mix containing 1×HiFi Buffer, 2.25 mM magnesium chloride, 0.3 μM of each forward and reverse primer, 1.2 mM deoxynucleotides and 0.5U KAPA HiFi DNA Polymerase. PCR conditions included one cycle of 95°C for 3 min, 30 cycles of 98°C 30 sec, 57°C 30 sec, 72°C 30 sec, and one cycle of 72°C for 5 min. Forward 5’-TCGTCGGCAGCGTCAGATGTGTATAAGAGACAGCCTACGGGNGGCWGCAG-3’ and reverse 5’-GTCTCGTGGGCTCGGAGATGTGTATAAGAGACAGGACTACHVGGGTATCTAATCC-3’ primers were based on previously optimized sequences [20].

### Library preparation

Amplicons, approximately 390 bp length were subjected to index PCR creating 460 bp libraries. These were pooled according to Illumina protocols [19] and sequenced on a MiSeq instrument using paired 300 bp reads and MiSeq v3 reagents (MS-102-3003, Illumina). The indices for each sample are provided in S1 Table.

### Microbiota analyses

Bioinformatic analyses of 16S rRNA gene data were carried out on demultiplexed fastq reads. Reads were processed with Skewer version 0.2.1 to filter out reads (Q < 12) and to remove adapter and primer sequences. Reads were analyzed with QIIME2 (version 2019.1.0) by implementing plug-ins including Deblur to denoise sequencing reads [21], SEPP to phylogenetically place reads [22], feature-classifier to taxonomically assign OTUs, UniFrac to compute beta-diversity analyses [23] and gneiss and balance trees [24] to examine relative abundances between variables. Further statistical tests were carried out in QIIME2 including pairwise PERMANOVA to compare beta-diversity and analysis of composition of microbiomes (ANCOM) to compare abundance of taxa between two populations. Prism (version 8.8.0) was used for comparison of sequence reads with Mann-Whitney test and calculation of alpha diversity significance with Wilcoxon matched-pairs signed rank test.

## Results

### Fecal microbiomes of *M*. *condylurus* in captivity have greater species richness than in the wild

**Fig 1:**
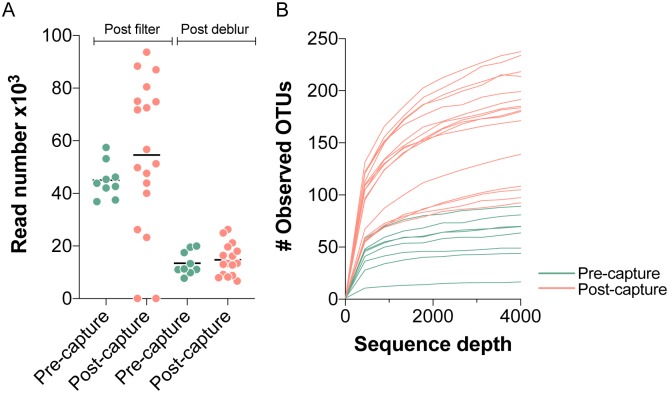
Number of sequencing reads and mapped OTUs in fecal bat samples. In **A** number of 16S rRNA V3-V4 gene reads passing read-trimming and quality filtering (post filter) and reads passing denoising (post Deblur) for bat fecal samples collected prior to capture (green) and post-capture (light-red) are shown for individual bats in a scatter plot. In **B** the number of observed OTUs recovered from data rarefied at a sequence depth of up to 4000 sequences are shown for each pre-capture and post-capture sample.

The number of 16S rRNA gene reads are shown post filter (pf) and post Deblur (pd) for each bat fecal sample in pre-capture (pf:45x10^3^ ± 6.7x10^3^ and pd:13.5 x10^3^ ± 4.4x10^3^) and post-capture (pf:54x10^3^ ± 28x10^3^ and pd:14x10^3^ ± 6.1x10^3^) groups in Fig 1A. Two samples that had sequence read numbers of < 100 in the post-capture group were removed from downstream analyses to reduce the influence of low read numbers on diversity metrics. The mean number of reads for pre-capture and post-capture groups are comparable (not significantly different Mann-Whitney test), although coefficient of variation in filtered and Deblur read numbers is high (14.9–51.5%), thus to reduce confounding effects of unequal read numbers on the analysis the data is subsampled at a depth of 4000 sequences. A subsampling depth of 4000 reads recovers the majority of OTUs for each sample as shown by transition to a plateau in the rarefaction curves at a sequencing depth of ~ 4000 presented in Fig 1B and this subsampling depth has been implemented to calculate various diversity metrics.

**Fig 2:**
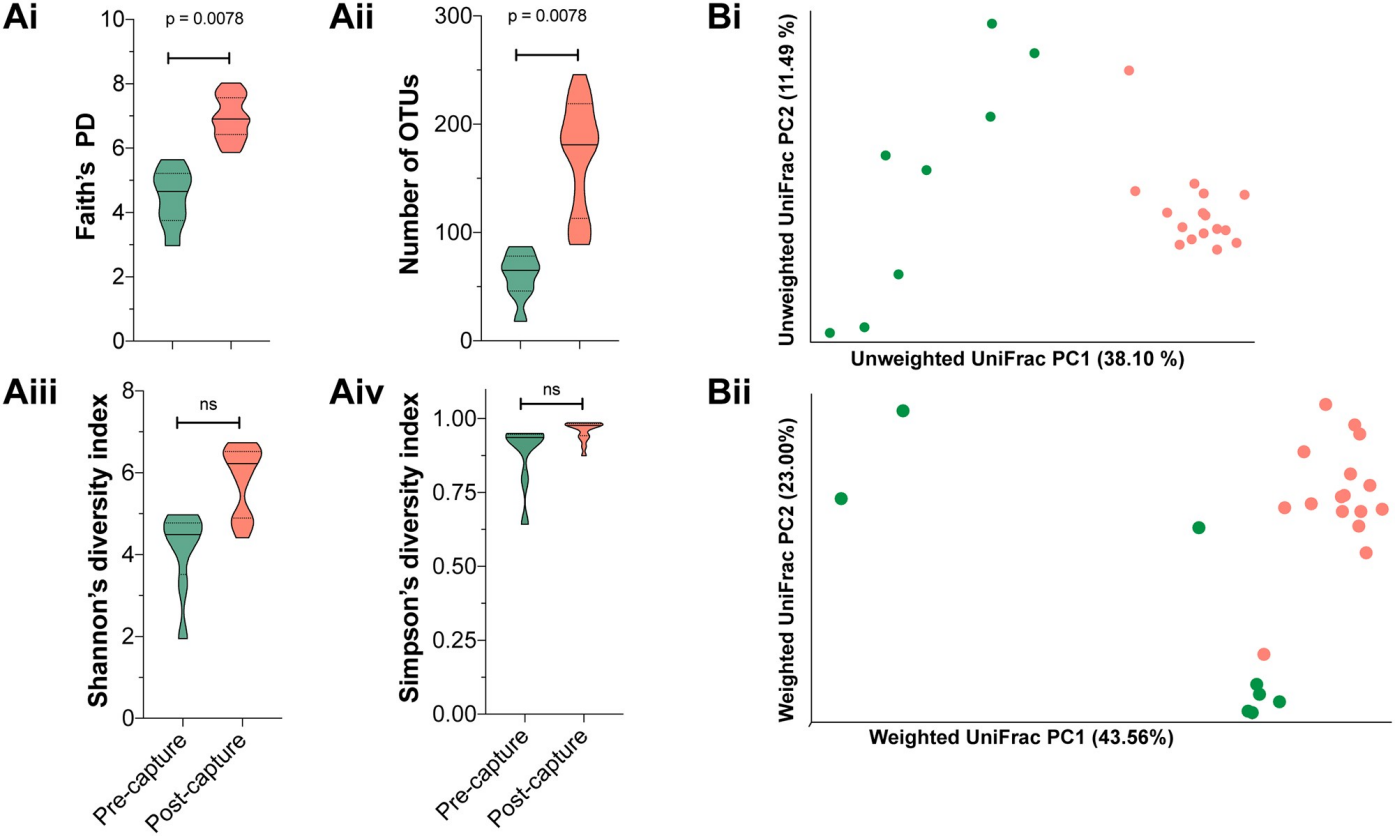
Alpha and beta diversity metrics for pre-capture and post-capture datasets. With violin plots several alpha diversity metrics are shown in **A** including: i. Faith’s phylogenetic diversity (PD) score ii. the number of OTUs iii. Shannon’s diversity index and iv. Simpson’s diversity index for OTU mapped 16S rRNA gene sequences amplified from pre-capture (green) and post-capture (light-red) fecal samples. The median and quartiles for the different scores are shown for nine pre-capture bats (green) and 16 post-capture bats (light-red). The beta diversity of microbial communities in each pre-capture (green) and post-capture (red) sample was calculated with unweighted UniFrac (**Bi**) and weighted UniFrac (**Bii**) and scaled values are shown with a PCoA plot visualized with Qiime2view.

Several alpha diversity metrics are calculated in QIIME2 to compare microbial diversity for individual bats within their pre-capture and post-capture microbiotas. Shown in Fig 2A these metrics determine the impacts of captivity on microbial species richness, diversity and evenness. Examining number of OTUs and Faith’s phylogenetic diversity (PD), which enumerates microbial richness by considering branch length and relatedness of the OTUs within the phylogenetic tree, reveal that post-capture microbiomes have greater species richness than pre-capture microbiomes (Fig 2Ai and 2Aii, p = 0.0078, Wilcoxon matched-pairs signed rank test). Shannon’s and Simpson’s indices, which take species abundance into account also tended to show higher diversity scores for post-capture samples, however this was not statistically significant (Fig 2Aiii and 2Aiv).

### Convergence of fecal microbiomes for microbats housed in captivity

Beta diversity metrics are used to measure the dissimilarity of microbial communities between samples. Beta diversity is calculated with QIIME2 by implementing unweighted- and weighted-UniFrac analyses and the results are shown in Fig 2Bi and 2Bii. Unweighted UniFrac data scaled with principal coordinates analysis (PCoA) reveals the first factor accounts for 38.1% of variation in the data (Fig 2Bi, **axis 1**). Post-capture samples form a distinct cluster on axis 1, while pre-capture samples disperse across the first and second axes. The difference observed between pre-capture and post-capture microbial communities on scaled PCoA plots is further supported by a significant difference between the two groups in unweighted UniFrac distance metrics (p<0.001, pseudo-F test statistic = 11.5, PERMANOVA) and weighted UniFrac distance metrics (p<0.001, pseudo-F test statistic = 9.3, PERMANOVA). Clustering of post-capture samples is also visible for analyses giving weight to microbial relatedness and microbial abundance, such as weighted UniFrac. In summary, these data reveal that post-capture, microbial species are significantly similar in taxonomic quality and abundance while at pre-capture the microbial communities are qualitatively diverse.

### Compositional changes associated with *M*. *condylurus* microbiome in captivity

**Fig 3:**
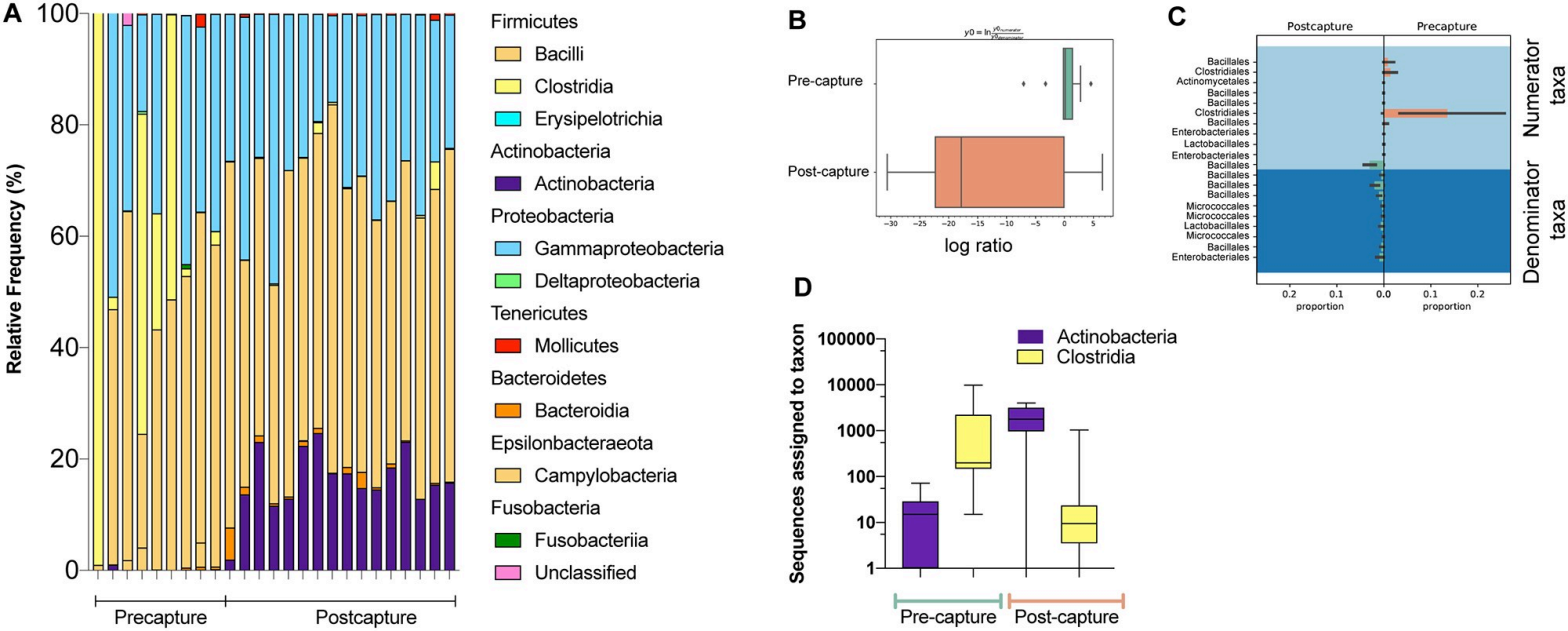
Taxonomical variation in abundance between fecal samples. The relative frequency of reads that mapped to classified features are shown in **A** at the class level for each bat sample collected pre-capture and post-capture. Feature abundance was clustered in reference to capture status and the log ratio is shown for balance y0 in **B**. The top 10 taxa at the level of order assigned to numerator or denominator with the largest positive and negative log fold changes are shown in **C**. The specific bacterial classes identified that were significantly different in ANCOM analysis and their sequence abundance in pre-capture and post-capture datasets (**D**).

The relative frequencies of taxa identified at the taxonomic level of bacterial class are shown for each pre-capture and post-capture sample in Fig 3A. The dominant bacterial phyla; class identified in most samples, regardless of capture status includes *Proteobacteria*; *Bacilli* and *Firmicutes*; *Gammaproteobacteria*. *Actinobacteria*; *Actinobacteria*, however, was mostly observed in post-capture samples only. To further address whether the abundance of these taxa contributed to the significant clustering of post-capture samples found in earlier UniFrac analyses, a deeper analysis of the changes in microbial composition between pre-capture and post-capture states was performed. This involved a balance tree analysis at the taxonomic level of order implemented with Gneiss in qiime2 [24].

Balance trees are used to interpret relative abundance by assessing community change with log ratios of geometric means of subtrees. Balance trees are not influenced by the fluctuation of non-overlapping taxa across variables, unlike statistical tests based on raw proportions, which may have inherent high levels of false-discovery and lead to statistical misinterpretation (18).

In this analysis we aim to identify differentially abundant taxa in the context of microbial community composition between pre-capture and post-capture groups. Initially the data is partitioned in clusters based on pairwise correlations in taxonomic abundance by implementing Ward’s hierarchical clustering method [25]. The clusters, which are portrayed on the heatmap’s y-axis (S1 Fig), form the balance tree and highlight the high-level structures in the data that are used to calculate each balance (y0-y9). Focusing on the balance identified with the greatest coefficient (13.2) and estimate of effect size for pre-capture/post-capture comparisons, y0, it was found that the log ratio is lower for the post-capture than the pre-capture group (Fig 3B). The analyses indicate taxa contributing to the y0 numerator such as *Clostridiales*, *Enterobacteriales* and *Actinomycetales* are at greater abundance in pre-capture samples, while taxa within the y0 denominator including *Micrococcales* and *Bacillales* are reduced in pre-capture samples relative to post-capture samples (Fig 3C). These changes in community composition were supported by results of an analysis of composition of microbiomes (ANCOM) at the taxonomic level of class that is shown in Fig 3D and portrays a greater number of sequences assigned to *Clostridia* across pre-capture samples (W = 11, violated null hypothesis of no difference between pre- and post-capture 11 times) and an increase in *Actinobacteria* assigned sequences in post-capture samples (W = 10). Overall, interrogation of the microbial composition has revealed the abundance of fecal microbiota changes in captivity in relation to the wild microbial community.

## Discussion

In this study, we investigated the influence of captivity on the fecal microbiota of microbats, *M*. *condylurus* by comparing microbial diversity in samples collected from bats on the day of capture and again from bats after six weeks of captivity. Using beta diversity metrics, we determined that the fecal microbial communities in pre-capture samples were phylogenetically diverse while those in post-capture samples were phylogenetically homogenous. For other mammals, such convergence of fecal microbial communities in captivity is largely explained by adaptation to a captive diet, residing in geographically close locations, and exposure to humans [10,26]. For bats, past studies have shown microbial composition is influenced by diet, environment, co-housing and feeding strategy [9,27,28]. One particular study by Xiao et al shows that convergence of the microbial fecal population, for various microbat species co-housed in a laboratory environment is due to dietary change over a six-month period [9]. One limitation of this study, and also our study here is that the microbial community of the ingested captive diet was not determined, and it is therefore unclear whether the fecal microbial species identified are commensal to the diet or are part of the native intestinal flora. Nevertheless, our study extends these findings by observing convergence during post-capture over a short time frame of six weeks. This has important implications for acclimatization time prior to embarking on experimental research with *M*. *condylurus* bats, which should be at least six weeks if the researcher intends to control for variations in microbial communities between different animals. On the other hand, if a wild microbiome is a prerequisite for a research outcome then performing research within six weeks after capture would be ideal. As we sampled bats from a single population, the acclimatization period is based on results from this particular bat colony. The period of microbiome convergence for microbats in disparate colonies may warrant further study to assess the generalizability of our findings.

Despite the homogeneity observed between post-capture samples, these same samples were also identified as more species rich (higher number of OTUs detected) than pre-capture samples with alpha diversity metrics. Capture status is the main factor affecting alpha diversity in this study. Other differences between individual bats (gender, mealworm consumption, body weight and time of capture), however, could influence microbiomes yet are unable to be determined due to small sample sizes. The effect of captivity on the bat microbiome does not correlate well with past research on species richness of bacterial communities in terrestrial mammals. This research found animals in the wild have greater microbial species richness than those kept in captivity and only in a small number of mammals (odd-toed ungulates) is the microbial community richness consistent irrespective of captive status [10,29]. Rather, this finding echoes those presented in bat specific studies which revealed species richness to increase in captivity for fruit bats [27] and microbats [9,30]. These studies have suggested post-capture species richness is a result of shared microbiomes and commensal bacteria ingested as part of the captive diet. One alternative explanation is a greater range of bacterial species can expand in the absence of bacteria required to process a diet rich in chitin, which forms part of the exoskeleton of insects that are consumed by *M*. *condylurus* and most microbats. Notwithstanding, prior studies have demonstrated a reduction in bacterial species richness in captive fruit bats [31] and other studies suggest inconsistencies in alpha diversity metrics between studies are common and result from different analysis pipelines, whereas beta diversity metrics may be more comparable between studies [32].

Microbial species classified within the *Firmicutes* and *Proteobacteria* phyla were commonly detected irrespective of capture status and this profile matches bat fecal microbiota profiles shown in other published works [9,17,28]. A balance trees analysis portrayed that the convergence of post-capture microbial landscapes was associated with unique increases in *Actinobacteria* spp. assigned to *Micrococcales* while the signature of pre-capture microbiomes was an abundance of *Clostridia* spp. The fluctuations in *Clostridia* spp. caused by captivity correspond to those seen in other captive mammals [10]. The uniqueness to our dataset is the identification of a high frequency of *Actinobacteria* spp. in captive bats, which is more typically identified at a lower frequency [33]. To understand the implications of finding *Actinobacteria* in abundance requires further interrogation into the commensal microbial communities of the captive diet as insects are known to harbor an abundance of *Proteobacteria* spp. but not *Actinobacteria* spp. in their gut [34].

Our findings reveal that fecal microbial communities in microbats change and converge as a result of captivity. Taken together, these findings imply that with the use of a short co-housing period of six weeks the fecal microbiome will become similar between disparate bats co-housed in captivity. A six-week acclimatization period could be implemented to reduce the confounding effects of inter-animal variation in microbial communities for a more controlled experimental system.

## Supporting information

S1 Fig Gneiss heatmap of Ward clustered partitions. Using the gneiss toolkit in Qiime2 a heatmap was created and shows the coefficient p values for balances y0-y9 after comparison of pre-capture and post-capture samples. Each balance consists of clustered OTUs that strongly correlate in their abundance in pre-capture or post-capture samples.
(PDF)

S1 Table Associated metadata with 16S rRNA gene sequence datasets. Gender, M (male) or F (female); Collection date, the date the bat fecal sample was taken; Index1 and Index2, paired-end indices used to demultiplex samples.
(DOCX)
