# Supplementary figures and images for: Microbiomes in the insectivorous bat species Mops condylurus rapidly converge in captivity
Source: PLoS One. 2020 Mar 20;15(3):e0223629. doi: 10.1371/journal.pone.0223629 (PMC7083271; doi:10.1371/journal.pone.0223629)

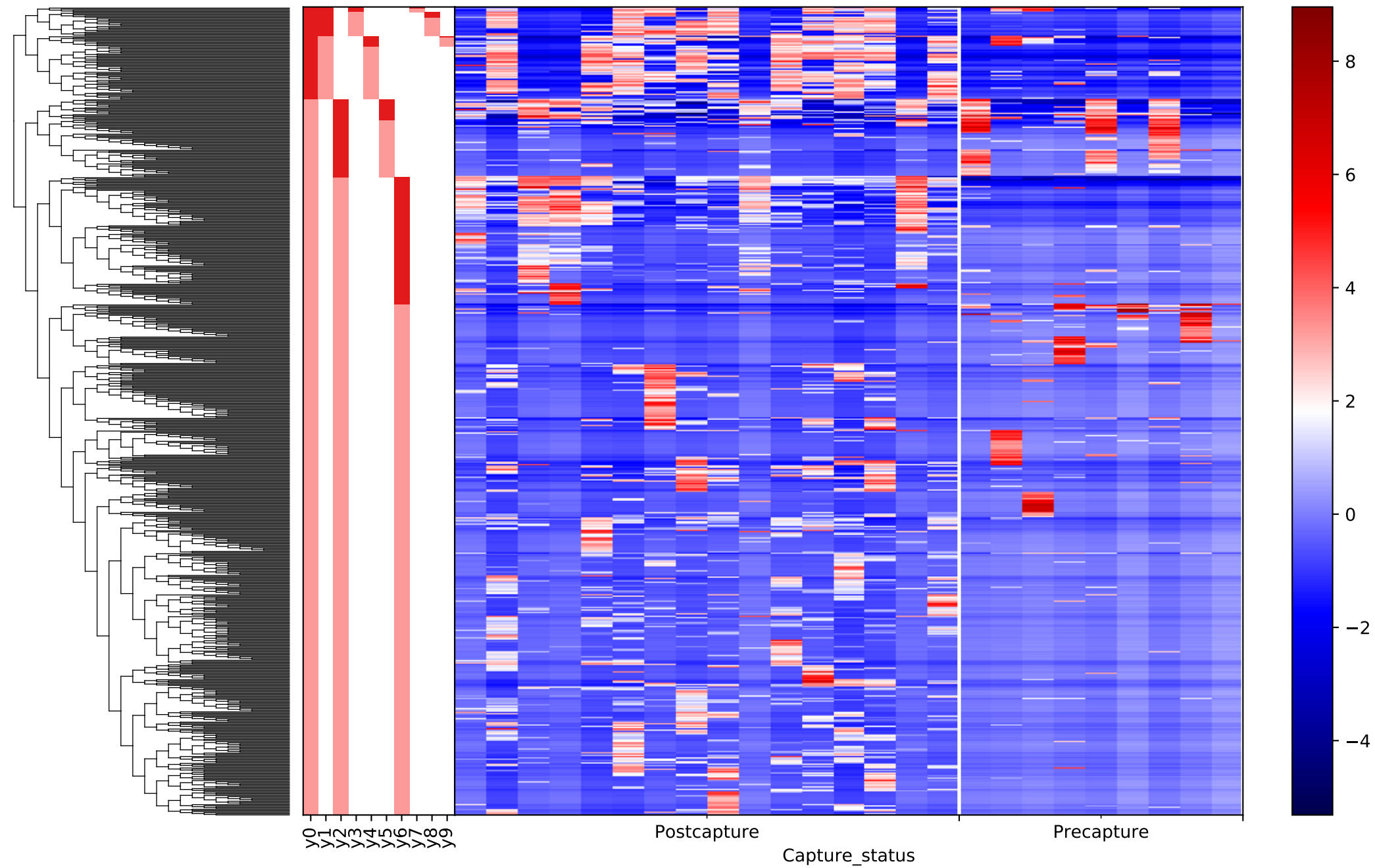

Supplement: S1 Fig — Using the gneiss toolkit in Qiime2 a heatmap was created and shows the coefficient p values for balances y0-y9 after comparison of pre-capture and post-capture samples. Each balance consists of clustered OTUs that strongly correlate in their abundance in pre-capture or post-capture samples. (PDF) [file pone.0223629.s001.pdf]
