# Supplementary material for: Microbiomes in the insectivorous bat species Mops condylurus rapidly converge in captivity
Source: PLoS One. 2020 Mar 20;15(3):e0223629. doi: 10.1371/journal.pone.0223629 (PMC7083271; doi:10.1371/journal.pone.0223629)
Supplement: S1 Table — Gender, M (male) or F (female); Collection date, the date the bat fecal sample was taken; Index1 and Index2, paired-end indices used to demultiplex samples. (DOCX) [file pone.0223629.s002.docx]

| Sample ID | Bat Number | Gender | Collection Date | Index1 | Index2 | Microbiome Status |
| --- | --- | --- | --- | --- | --- | --- |
| 1 | 1 | M | 7/4/17 | ACTCGCTA | CGTCTAAT | Pre-capture |
| 2 | 3 | F | 7/4/17 | ACTCGCTA | TCTCTCCG |  |
| 3 | 8 | F | 7/4/17 | GGAGCTAC | CTCTCTAT |  |
| 4 | 24 | M | 14/4/17 | GGAGCTAC | TATCCTCT |  |
| 5 | 25 | M | 14/4/17 | GGAGCTAC | GTAAGGAG |  |
| 6 | 26 | M | 14/4/17 | GGAGCTAC | ACTGCATA |  |
| 7 | 28 | M | 22/4/17 | GGAGCTAC | AAGGAGTA |  |
| 8 | 34 | M | 23/4/17 | GGAGCTAC | CTAAGCCT |  |
| 9 | 35 | F | 23/4/17 | GGAGCTAC | CGTCTAAT |  |
| 10 | 1 | M | 19/5/17 | GCGTAGTA | GTAAGGAG | Post-capture |
| 11 | 3 | F | 19/5/17 | GCGTAGTA | ACTGCATA |  |
| 12 | 8 | F | 19/5/17 | GCGTAGTA | AAGGAGTA |  |
| 13 | 13 | F | 19/5/17 | GCGTAGTA | CTAAGCCT |  |
| 14 | 14 | M | 19/5/17 | GCGTAGTA | CGTCTAAT |  |
| 15 | 15 | F | 19/5/17 | CGGAGCCT | CTCTCTAT |  |
| 16 | 18 | M | 19/5/17 | CGGAGCCT | TATCCTCT |  |
| 17 | 24 | M | 19/5/17 | CGGAGCCT | GTAAGGAG |  |
| 18 | 25 | M | 19/5/17 | CGGAGCCT | ACTGCATA |  |
| 19 | 26 | M | 19/5/17 | CGGAGCCT | AAGGAGTA |  |
| 20 | 28 | M | 19/5/17 | CGGAGCCT | CTAAGCCT |  |
| 21 | 33 | M | 19/5/17 | CGGAGCCT | CGTCTAAT |  |
| 22 | 34 | M | 19/5/17 | CGGAGCCT | TCTCTCCG |  |
| 23 | 35 | F | 19/5/17 | TACGCTGC | CTCTCTAT |  |
| 24 | 41 | M | 19/5/17 | TACGCTGC | TATCCTCT |  |
| 25 | 49 | M | 19/5/17 | TACGCTGC | GTAAGGAG |  |
| 26 | 50 | F | 19/5/17 | TACGCTGC | ACTGCATA |  |

**S1 Table**
